# Physical activity and sedentary time across pregnancy and associations with neonatal weight, adiposity and cord blood parameters: a secondary analysis of the DALI study

**DOI:** 10.1038/s41366-023-01347-9

**Published:** 2023-07-27

**Authors:** Anna M. Dieberger, Barbara Obermayer-Pietsch, Jürgen Harreiter, Gernot Desoye, Mireille N. M. van Poppel, DALI Core Investigator group, David Simmons, Rosa Corcoy, Juan M. Adelantado Perez, Alexandra Kautzky-Willer, Peter Damm, Elizabeth Mathiesen, Dorte M. Jensen, Lise Lotte T. Andersen, Fidelma Dunne, Annunziata Lapolla, Maria G. Dalfra, Alessandra Bertolotto, Judith G. M. Jelsma, Sander Galjaard, Ewa Wender-Ozegowska, Agnieszka Zawiejska, David Hill, Roland Devlieger, Frank J. Snoek

**Affiliations:** 1 Department of Obstetrics and Gynaecology, Medical University of Graz, Graz, Austria; 2 Division of Endocrinology and Diabetology, Endocrinology Lab Platform, Department of Internal Medicine and Department of Obstetrics and Gynaecology, Medical University of Graz, Graz, Austria; 3 Division of Endocrinology and Metabolism, Department of Internal Medicine III, Medical University of Vienna, Vienna, Austria; 4 Institute of Human Movement Science, Sport and Health, University of Graz, Graz, Austria; 5 Macarthur Clinical School, School of Medicine, Western Sydney University, Campbelltown, NSW Australia; 6 Institut de Recerca de l’Hospital de la Santa Creu i Sant Pau, Barcelona, Spain; 7 CIBER-BBN, ISCIII, Madrid, Spain; 8 Gender Institute Lapura, Gars am Kamp, Austria; 9 Department of Obstetrics and Center for Pregnant Women with Diabetes, Rigshospitalet, Copenhagen, Denmark; 10 Department of Clinical Medicine, University of Copenhagen, Copenhagen, Denmark; 11 Department of Endocrinology and Center for Pregnant Women with Diabetes,Rigshospitalet, Copenhagen, Denmark; 12 Department of Endocrinology and Department of Gynecology and Obstetrics, Odense University Hospital, Odense, Denmark; 13 Department of Clinical Research, Faculty of Health Sciences, University of Southern Denmark, Odense, Denmark; 14 Clinical Research Facility and College of Medicine Nursing and Health Sciences, University of Galway, Galway, Ireland; 15 Università Degli Studi di Padova, Padua, Italy; 16 Azienda Ospedaliero Universitaria Pisa, Pisa, Italy; 17 Department of Public and Occupational Health, Amsterdam UMC, Vrije Universiteit Amsterdam, Amsterdam, The Netherlands; 18 Amsterdam Public Health Research Institute, Health Behaviors & Chronic Diseases, Amsterdam, The Netherlands; 19 Department of Obstetrics and Gynaecology, Division of Obstetrics and Prenatal Medicine, Erasmus MC, University Medical Centre Rotterdam, Rotterdam, The Netherlands; 20 Medical Faculty I, Poznan University of Medical Sciences, Poznan, Poland; 21 Lawson Health Research Institute, London, ON Canada; 22 KU Leuven Department of Development and Regeneration: Pregnancy, Fetus and Neonate, Gynaecology and Obstetrics, University Hospitals Leuven, Leuven, Belgium; 23 Department of Medical Psychology, Amsterdam UMC, Vrije Universiteit Amsterdam, Amsterdam, The Netherlands

**Keywords:** Paediatrics, Public health, Obesity, Epidemiology

## Abstract

**Background/Objectives:**

Obesity during pregnancy is associated with neonatal adiposity, which is a risk factor for childhood obesity. Maternal physical activity (PA) and sedentary behaviours during pregnancy might modify this risk. We therefore studied associations between maternal PA and sedentary time (ST) during pregnancy and neonatal anthropometry and cord blood parameters and investigated whether associations differed by offspring sex.

**Subjects/Methods:**

Participants of the Vitamin D And Lifestyle Intervention for Gestational Diabetes Mellitus Prevention (DALI) study with a BMI  ≥ 29 kg/m^2^ were analysed as a cohort. Maternal moderate-to-vigorous PA (MVPA) and ST were measured repeatedly with accelerometers across pregnancy. Associations between mean levels and changes in MVPA and ST and birthweight, neonatal adiposity (fat mass (FM)%) and cord blood parameters, including C-peptide, leptin and lipids, were analysed in 213 mother-child pairs with Bayesian multilevel models. Interactions with offspring sex were considered.

**Results:**

Almost all women decreased MVPA levels and increased ST throughout gestation. Both higher maternal mean MVPA and increasing MVPA were associated with lower offspring FM% in males (−0.520%; 95% CI: −1.011%, −0.031% and −4.649%; −7.876%, −1.432% respectively). In female offspring, mean ST was associated with lower cord blood C-peptide (−0.145 µg/l; −0.279 µg/l, −0.005 µg/l). No associations were found with birthweight or other cord blood parameters.

**Conclusions:**

Maternal MVPA is associated with neonatal fat mass, but not birthweight, in male offspring. Our findings underline the importance of physical activity throughout pregnancy.

## Introduction

Concurrent with the global obesity epidemic, childhood obesity rates are on the rise [1]. Children are increasingly large at birth, with birthweight and numbers of macrosomic and LGA (large-for-gestational age) neonates increasing [2, 3]. These children then tend to stay large, as macrosomia and LGA are known risk factors for childhood obesity [4]. This association might be explained by the increased fat mass in those with higher birthweight, as neonatal adiposity (i.e. increased fat mass) is associated with childhood overweight and obesity [5, 6].

One major contributor to high birthweight and neonatal adiposity is maternal obesity [7, 8]. To break this inter-generational cycle of obesity, it is imperative to find strategies to attenuate the risk of macrosomia/LGA and neonatal adiposity and consequently childhood obesity. As factors such as obesity are difficult to change once women are pregnant, it is important to investigate other modifiable lifestyle factors such as physical activity (PA) and sedentary behaviours in relation to neonatal weight and body composition, in particular neonatal fat mass.

A recent meta-analysis showed that PA interventions in pregnancy reduce the risk of LGA neonates, without increasing numbers of small-for-gestational age (SGA) neonates [9]. In contrast, a cohort study found that mid-pregnancy PA was associated with reduced neonatal adiposity but not with lower birthweight [10]. Another cohort study also found that mothers who frequently engaged in moderate PA in early pregnancy gave birth to neonates with a lower fat mass, but this reduction was only found in neonates at the highest centiles of fat mass [11].

In our own previous work as part of the DALI (Vitamin D And Lifestyle Intervention for Gestational Diabetes Mellitus Prevention) study, a lifestyle intervention, combining counselling on PA and healthy eating during pregnancy, did not affect birthweight [12], but did reduce neonatal adiposity. This effect was mediated by a reduction in self-reported maternal sedentary time (ST) [13]. Thus, in literature, associations between PA and either birthweight or neonatal adiposity have been consistently shown. However, it is unclear what the influence of differences in timing of PA in pregnancy is, and which exact body compartment is affected (total weight, fat mass). In addition, there is a lack of literature on the association of sedentary behaviour (independent from PA) with neonatal adiposity. Furthermore, many previous studies used subjective, self-reported physical activity measurements [8, 10, 11, 13], making results less valid. In addition, many cohort studies measured PA levels only once, thereby making it impossible to determine potential effects of changes in activity levels across pregnancy.

In previous studies, relevant sex-interactions were observed for associations of maternal metabolic parameters and neonatal outcomes [14, 15], and male neonates might be more susceptible to changes in nutrient supply [16, 17]. Based on literature, we hypothesise that more maternal PA in pregnancy is associated with reduced neonatal adiposity, and more sedentary behaviour with increased adiposity, and that these associations are sex-specific. Therefore, the aim of this study was to examine the association between device-based, longitudinally measured PA and ST in pregnancy and neonatal adiposity and birthweight, taking sex differences into account. In addition, to examine potential underlying pathways, the association between PA and ST and cord blood parameters was also investigated.

## Methods

### Study population

This is a secondary analysis of the DALI study, a randomised controlled trial, which was preceded by a pilot study. The trial was set up in eleven different centres in nine European countries (Austria, Belgium, Denmark (Odense, Copenhagen), Ireland, Italy (Padua, Pisa), Netherlands, Poland, Spain and United Kingdom). The study was registered under trial registration number ISRCTN70595832. All local ethics committees provided ethical approval and written informed consent was signed by all participants prior to data collection [18].

Women included in the study were aged ≥18 years, <20 weeks of gestation with a singleton pregnancy and had a pre-pregnancy body mass index (BMI) of ≥29 kg/m^2^. All women were subjected to an oral glucose tolerance test (OGTT) and screened for gestational diabetes mellitus (GDM) before 20 weeks of gestation, and those diagnosed with GDM according to International Association of Diabetes in Pregnancy Study Groups (IADPSG) criteria [19] were excluded. Women with pre-existing diabetes or other chronic medical diseases were also excluded. Abnormal calcium metabolism or calcium measurements in early pregnancy were exclusion criteria for the vitamin D trial.

For the primary study, participants of the pilot study were randomised to healthy eating (HE) or PA counselling, or a combination of both (HE&PA). In the lifestyle trial, HE, PA or HE&PA counselling interventions were compared to a control group. In the vitamin D trial, vitamin D supplementation with and without HE&PA counselling were compared to a placebo group, with or without the HE&PA intervention. For this secondary analysis, all randomised participants from the pilot, lifestyle and vitamin D study were combined into one cohort and data analysed accordingly in an observational manner.

Only mother-child dyads that had at least one neonatal outcome measure (cord blood or skinfold measurements) available, were included. In addition, to allow for longitudinal analyses during pregnancy, at least two out of three physical activity measurements had to be available.

### Measurements

Data were collected at three times during pregnancy (<20 weeks, 24–28 weeks, 35–37 weeks), and after delivery. To assess whether study participants developed GDM while participating in the study, all women were again subjected to an OGTT at the second and third visit according to IADPSG criteria. Maternal characteristics including age, ethnicity, parity, marital and employment status, smoking and alcohol intake, and pre-pregnancy weight were collected by questionnaire. Height was measured during the first prenatal visit on a stadiometer (SECA 206, SECA, Birmingham, UK). Weight was measured at each prenatal visit using calibrated electronic scales (SECA Measure 888; 887). Pre-pregnancy BMI was calculated as pre-pregnancy weight (kg) divided by the square of height (m^2^). Information on offspring sex, gestational age at birth and mode of delivery was extracted from medical records.

#### Physical activity measurements

Physical activity data were measured with accelerometers (ActiGraph GTM1, GT3X+ or Actitrainer; Pensacolada, Florida, USA) three times during pregnancy (<20 weeks, 24–28 weeks, 35–37 weeks). Participants were asked to wear the device on an elastic belt positioned over the right hip during waking hours for at least 3 days. Hip-worn accelerometers have been shown to provide similar estimates to wrist-worn devices [20] and appear to be less influenced by walking style when worn on the hip compared to wrist-worn accelerometers [21]. The accelerometer had to be removed during showering, bathing or swimming and participants were asked to record the time and reason of removal in an activity diary, which was used to clean data manually. Data were recorded in 1-min epochs. Non-wear time was defined as periods of consecutive strings of zero counts for at least 90 min [22]. Per time point, at least 480 min per day on at least three valid days had to be available or data were coded as missing. Average minutes per day spent sedentary (<100 counts/min), in light (100–1951 counts/min) and moderate-to-vigorous physical activity (MVPA) (<1951 counts/min) were calculated according to Freedson cut-offs [23]. Time spent swimming as recorded in the activity diary was added as minutes spent in MVPA [23, 24].

#### Laboratory analyses

Immediately after delivery, venous cord blood samples were taken and stored at −20 °C or colder until further analysis in the central trial laboratory in Graz, Austria. Leptin was quantified by solid-phase sandwich ELISA (E05-086-96; EIASON, Graz, Austria) with an analytical sensitivity of 1.0 ng/ml, an intra-assay coefficient of variability of 6.0–6.9% and an inter-assay coefficient of variability of 8.7–11.6%. C-peptide was quantified by chemiluminometric solid-phase sandwich immune assay (ADVIA Centaur; Siemens Healthcare Diagnostics, Vienna, Austria). Analytical sensitivity was 0.05 ng/ml and intra-assay coefficient of variability was 3.7–4.1%, inter-assay coefficient of variability was 6.1–6.2%. 3-β-hydroxybutyrate (3BHB), total cholesterol (TC) and triglycerides (TG) were measured using colorimetric enzymatic assays, using DiaSys Diagnostic Systems (Holzheim, Germany) reagents and were calibrated using secondary standards from DiaSys Diagnostics for 3BHB and Roche Diagnostics (Mannheim, Germany) for TC and TG. HDL cholesterol (HDL-C) was measured with a homogenous assay from DiaSys Diagnostics. LDL cholesterol (LDL-C) was calculated according to the Friedewald formula (LDL-C = TC – HDL-C – TG/5) [25]. Non-esterified fatty acids (FFAs) were analysed using an enzymatic reagent and standards from Wako Chemicals (Neuss, Germany). All lipid analyses were performed on an Olympus AU640 automatic analyser (Beckman Coulter, Brea, CA). All assays were performed according to the manufacturer’s instructions.

#### Neonatal anthropometry

Birthweight was measured at birth. As indicator of neonatal adiposity, subcutaneous skinfold thickness was measured in mm within 48 h of birth with a Harpenden skinfold calliper (British Indicators, Sussex, England) at four sites (triceps, subscapular, supra-iliac and quadriceps) [18]. Each measurement was taken twice, and the mean value calculated. If a difference of more than 0.2 mm was registered, the measurement was repeated a third time and the average of the three was taken. Age of the neonates at the time of the measurement was reported in hours.

The sum of the four skinfold measurements (SSF) was calculated. Additionally, fat mass in kg was estimated, according to a validated equation, including an adjustment for neonatal age at SSF measurement in hours [26]. Estimated fat mass percentage (FM%) was calculated by dividing the estimated fat mass by total body mass multiplied by 100.

### Statistical analysis

Maternal and neonatal characteristics are presented as count and proportion and mean and standard deviation (SD), or median and interquartile range (IQR) for skewed data. Normal distribution was assessed visually by histograms. Neonatal characteristics were split up by sex and tested by *χ*^2^ tests or unpaired *t*-tests. Skewed variables were tested by Mann–Whitney *U* tests, as log-transformation was not possible due to some variables containing the value zero.

The average daily time spent in MVPA and ST is presented in minutes and as proportions of daily accelerometer wear time per time point. Daily wear time is presented in minutes. Variables are presented as mean and SD or median and IQR and tested by paired sample *t*-test; skewed variables were log-transformed beforehand.

#### Longitudinal analyses

To analyse the relationship between the longitudinally, repeatedly measured independent variables (maternal MVPA and ST) and the fixed neonatal dependent variables (neonatal anthropometry, cord blood parameters) measured at a later time point, a two-step analysis was performed as previously described by Welten et al. [27]. For all longitudinal analyses, ST was used as proportion of daily wear time, as ST, but not MVPA, is highly dependent on accelerometer wear time. Time spent in light physical activity was not added to the models, as this is automatically factored in by having both MVPA and ST in the model. For example, a reduction in daily ST without a change in minutes spent in MVPA automatically means an increase in light PA, as a person’s total day is made up of the minutes spent in ST, light PA and MVPA.

In the first step, a multilevel analysis was performed with gestational age (weeks) at the PA measurement as independent variable and the repeated measurements of MVPA (min/day) or ST (% daily wear time) as dependent variable on level one, nested within the individuals on level two, including a random intercept and random slope. Thereby, individual slopes, representing the individual change in MVPA or ST over the course of pregnancy, were calculated for each participant. These individual MVPA and ST slopes were then exported as two new variables (MVPA: change min/week; ST: change %/week). In addition, for each participant, individual averages of ST and MVPA over the course of pregnancy were calculated. The individual mean values and slopes formed the independent variables for step two. To increase readability of estimates, mean MVPA and ST were transformed into units of 10 min and 10% wear time respectively.

In the second step, another multilevel analysis was performed with the individuals on level one nested within the different study centres on level two, including a random intercept. The mean and slope variables of MVPA and ST were added into the model as independent variables and analyses were performed for each dependent variable (cord blood parameters, neonatal FM% and birthweight). The analyses were adjusted for the following a priori selected covariates: maternal smoking (yes/no), educational level (low/medium/high), parity (multiparity/nulliparity), pre-pregnancy BMI (kg/m^2^), gestational age at birth and randomisation group (HE yes/no, Vit D yes/no). Analyses with cord blood were additionally adjusted for mode of delivery (spontaneous/assisted vaginal/caesarean section). The variables gestational age at PA measurements and at birth and maternal pre-pregnancy BMI were centred around the mean for multilevel analyses.

Offspring sex was considered an effect modifier by adding interaction terms of each MVPA and ST variable and sex to the model. If interaction terms were significant (within 90% credible interval (CI)), analyses were subsequently repeated for male and female offspring separately.

Two sensitivity analyses were performed: In some neonates (*n* = 15), body composition was measured more than 48 h after birth. Neonatal FM% analyses were therefore repeated, including only those measured within 48 h after birth. Secondly, analyses for neonatal FM% were repeated with SSF as outcome instead, with neonatal age at time of measurement as additional covariate.

Due to the relatively small sample size, linear Bayesian multilevel models were used instead of maximum likelihood-based models. Weakly informative priors were used. Effective sample size, autocorrelation, R-hat and comparisons of the observed and predicted posterior distribution were inspected individually for each model to determine model fit and convergence. The default distribution of the response variable was Gaussian. Alternatively, if model fit was not satisfactory, a skewed normal or logarithmic normal distribution was selected.

A *p* value of <0.05 or results within the 95% CI were deemed significant. All analyses were performed in R: A language and environment for statistical computing (version 4.1.2) [28]. Bayesian multilevel analyses were performed using the brms package (version 2.16.3) [29], which uses Stan on the back-end (version 2.21.3) [30]. Plots were produced using the ggplot2 package (version 3.3.5) [31].

## Results

### Study participants

**Fig. 1:**
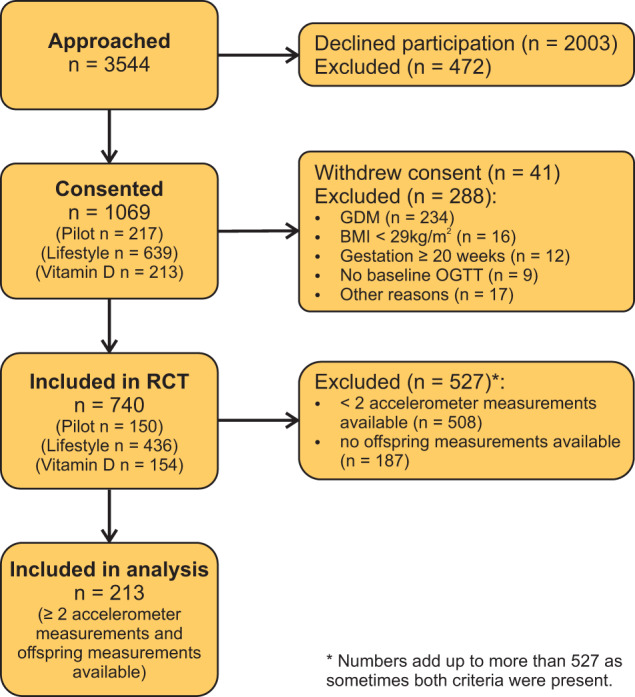
Flow chart of included participants. BMI body mass index, GDM gestational diabetes mellitus, OGTT oral glucose tolerance test, RCT randomised controlled trial.

Figure 1 depicts the flow chart describing participant recruitment and exclusions throughout the study. Of the 740 women enrolled in the RCT, 213 mother-child pairs had ≥2 accelerometer measurements and at least one neonatal outcome measured and were included in the analyses.

The most common reasons for participants not supplying accelerometer data were no device being available, women dropping out of the study or declining to wear the device, and at the last time point, having already delivered. In 10% of the cases, women who had received an accelerometer did not provide data due to an empty battery, a device being lost or not correctly initialised, or women not wearing the accelerometer. In 12% of cases data were invalid because insufficient wear time was recorded.

No participants from the study site Pisa, Italy, were included as they did not have any cord blood or skinfold measurements available. Participants from Padua, Italy only provided cord blood data, but no skinfold measurements.

Maternal characteristics are presented in Table 1. The majority of participants were Caucasians (80.8%), living with a partner (93.9%) and had a median BMI of 32.6 kg/m^2^. In total, 32.4% of the participants developed GDM after inclusion.

**Table 1:** Maternal characteristics.

	*n*	
Age, years (mean ± SD)	213	32.7 ± 5.3
Parity, multiparous (*n* (%))	213	110 (51.6)
Pre-pregnancy BMI, kg/m^2^ (median (IQR))	213	32.6 (30.8, 35.2)
Ethnicity, Caucasian (*n* (%))	213	172 (80.8)
Smoking (*n* (%))	213	29 (13.6)
Alcohol intake (*n* (%))	211	11 (5.2)
Education (*n* (%))	213	
Low		25 (11.7)
Medium		70 (32.9)
High		118 (55.4)
Marital status, with partner (*n* (%))	213	200 (93.9)
GDM diagnosis at 24–28 or 35–37 weeks (*n* (%))	204	66 (32.4)
GWG at 35–37 weeks, kg (mean ± SD)	206	8.1 ± 5.0

*BMI* body mass index, *GDM* gestational diabetes mellitus, *GWG* gestational weight gain, *IQR* interquartile range, *SD* standard deviation.

Male neonates were heavier at birth and had lower FM% compared to females (Table 2). Skinfolds were not significantly different between male and female neonates. Females had higher cord blood leptin, TC and LDL-C compared to males. Other cord blood parameters did not differ by sex.

**Table 2:** Neonatal characteristics by offspring sex.

	*n*	Males (*n* = 110)	*n*	Females (*n* = 102)
Gestational age at birth, weeks (median (IQR))	110	40.0 (39.0, 41.3)	102	39.7 (38.9, 40.4)
Mode of delivery (*n* (%))	110		101	
Spontaneous		59 (53.6)		56 (55.4)
Assisted vaginal delivery		17 (15.5)		12 (11.9)
Caesarean section		34 (30.9)		33 (32.7)
Anthropometry				
Birthweight, g (mean ± SD)	110	3606 ± 507	102	3462 ± 479^a^
Estimated fat % (mean ± SD)	99	12.2 ± 3.5	94	13.8 ± 3.4^a^
Triceps skinfold, mm (median (IQR))	102	4.7 (3.9, 6.1)	97	4.9 (4.2, 5.8)
Subscapular skinfold, mm (median (IQR))	101	4.5 (3.8, 5.4)	97	4.8 (4.1, 5.3)
Flank skinfold, mm (median (IQR))	102	3.8 (3.1, 4.8)	97	4.0 (3.4, 4.7)
Thigh skinfold, mm (median (IQR))	102	6.2 (5.3, 7.5)	97	6.6 (5.7, 7.9)
SSF, mm (median (IQR))	101	19.8 (16.7, 22.8)	97	20.3 (18.2, 23.4)
Cord blood parameters				
C-peptide, µg/l (median (IQR))	78	0.70 (0.52, 0.87)	74	0.69 (0.44, 0.92)
Leptin, µg/l (median (IQR))	77	7.40 (4.09, 11.10)	72	9.59 (5.88, 15.45)^a^
3BHB, mmol/l (median (IQR))	86	0.20 (0.10, 0.30)	74	0.20 (0.10, 0.30)
TC, mmol/l (median (IQR))	86	1.40 (1.24, 1.68)	74	1.63 (1.37, 1.98)^a^
HDL-C, mmol/l (median (IQR))	66	0.52 (0.41, 0.70)	58	0.59 (0.47, 0.72)
LDL-C, mmol/l (median (IQR))	66	0.69 (0.54, 0.87)	58	0.79 (0.61, 1.01)^a^
FFA, mmol/l (median (IQR))	66	0.29 (0.17, 0.46)	58	0.28 (0.19, 0.43)
TG, mmol/l (median (IQR))	86	0.42 (0.29, 0.55)	74	0.40 (0.28, 0.55)

*3BHB* 3-β-hydroxybutyrate, *FFA* free fatty acids, *HDL-C* high-density lipoprotein cholesterol, *IQR* interquartile range, *LDL-C* low-density lipoprotein cholesterol, *SD* standard deviation, *SSF* sum of skinfolds, *TC* total cholesterol.

^a^Significant difference between male and female offspring (*p* < 0.05).

### Changes in MVPA and ST across gestation

Median MVPA levels were significantly lower at 35–37 weeks of gestation, compared to measurements at <20 weeks and 24–28 weeks of gestation (Table 3). While ST in min/day did not significantly change, when adjusted for wear time, the proportion of daily time spent sitting increased from early gestation (<20 weeks) to 24–28 weeks by 1.8% corresponding to around 15 min more ST calculated based on a daily wear time of around 800 min. It did not significantly increase further until the end of pregnancy (Table 3). Accelerometer wear time reduced across pregnancy from an average of 830 min/day in the first half of pregnancy to 791 min/day at 35–37 weeks of gestation.

**Table 3:** MVPA and ST across pregnancy.

	<20 weeks (*n* = 187)	24–28 weeks (*n* = 180)	35–37 weeks (*n* = 164)
MVPA, min/day (median (IQR))	38.2 (24.5, 53.2)	34.1 (21.5, 50.0)	29.6 (16.2, 43.5)^a,b^
MVPA, % wear time (median (IQR))	4.5 (2.9, 6.3)	4.0 (2.7, 6.5)	3.7 (2.2, 5.1)^a,b^
ST, min/day (mean ± SD)	577.9 ± 100.3	578.0 ± 105.2	575.8 ± 96.3
ST, % wear time (mean ± SD)	69.6 ± 9.3	71.4 ± 9.4^a^	72.8 ± 8.1^a^
Accelerometer wear time, min/day (mean ± SD)	830.2 ± 87.8	807.0 ± 90.4^a^	791.3 ± 96.6^a,b^

*IQR* interquartile range, *MVPA* moderate-to-vigorous physical activity, *SD* standard deviation, *ST* sedentary time.

^a^Significantly different compared to <20 weeks (*p* < 0.05).

^b^Significantly different compared to 24–28 weeks (*p* < 0.05).

### Estimated individual changes in MVPA and ST across gestation

**Fig. 2:**
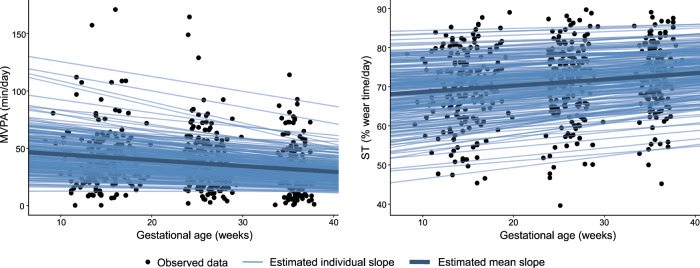
Estimated individual changes in MVPA and ST across gestation. MVPA moderate-to-vigorous physical activity, ST sedentary time. Estimated individual slopes represent the individually estimated changes in MVPA/ST across gestation, which are summarised as the estimated mean slope. Nearly all participants (212/213) have a negative MVPA slope, meaning they decreased their MVPA levels across gestation (mean MVPA slope = −0.51 min/week; 95% CI: −0.69 to −0.32 min/week). Out of 213 participants, 209 have a positive ST slope, meaning they increased their ST across gestation (mean ST slope = 0.16 %/week; 95% CI: 0.10 to 0.22 %/week).

Figure 2 shows the mean and individual estimated slopes of MVPA and ST across gestation. The slopes represent the estimated change in MVPA and ST over the course of pregnancy. The majority of participants decreased their MVPA (mean change −0.51 min/week) and concurrently increased their ST by on average 0.16% per week with increasing gestation. This corresponds to a decrease of 15 min in MVPA and a 40-min increase of daily ST within the period covered in the study (from week 10 to 40 of gestation), based on a daily wear time of around 800 min.

### Associations between maternal MVPA and ST and neonatal outcomes

Table 4 presents the associations between MVPA and ST means and slopes and neonatal anthropometry and cord blood parameters. Women who on average participated in higher levels of MVPA during pregnancy had offspring with lower FM% (−0.426%; 95% CI: −0.794%, −0.053%). This effect was sex-specific: both higher mean MVPA and an increase in MVPA were associated with lower neonatal FM% in males (−0.520%; 95% CI: −1.011%, −0.031% and −4.649%; 95% CI: −7.876%, −1.432%, respectively), while no significant associations were found in female offspring.

**Table 4:** Associations between maternal MVPA and ST and neonatal anthropometrics and cord blood parameters.

	Mean MVPA (10 min)	Slope MVPA (min/week)	Mean ST (10% wear time)	Slope ST (% wear time/week)
	Estimate (95% CI)	Estimate (95% CI)	Estimate (95% CI)	Estimate (95% CI)
Anthropometrics				
Neonatal FM%	**−0.426 (−0.794; −0.053)**	−2.244 (−4.624; 0.165)	−0.336 (−1.175; 0.501)	−4.527 (−11.901; 2.940)
Males	**−0.520 (−1.011; −0.031)**	**−4.649 (−7.867; −1.432)**	0.717 (−0.431; 1.844)	−4.688 (−14.649; 5.305)
Females	−0.334 (−0.914; 0.218)	−0.246 (−3.736; 3.262)	−0.842 (−2.079; 0.308)	−2.870 (−14.179; 8.206)
Birthweight, kg	−0.043 (−0.093; 0.004)	−0.243 (−0.563; 0.078)	−0.060 (−0.160; 0.042)	−0.526 (−1.480; 0.472)
Cord blood parameters				
C-peptide, µg/l	0.016 (−0.026; 0.060)	0.108 (−0.214; 0.454)	−0.061 (−0.143; 0.031)	−0.466 (−1.364; 0.477)
Males	0.025 (−0.026; 0.072)	0.225 (−0.148; 0.628)	0.020 (−0.087; 0.127)	0.230 (−1.130; 1.640)
Females	0.047 (−0.026; 0.118)	0.289 (−0.247; 0.853)	**−0.145 (−0.279; −0.005)**	−0.942 (−2.263; 0.445)
Leptin, µg/l	0.135 (−0.512; 0.765)	4.936 (−0.693; 10.737)	−0.466 (−1.745; 0.799)	7.791 (−4.598; 19.915)
3BHB, mmol/l	−0.017 (−0.034; 0.001)	−0.053 (−0.179; 0.085)	−0.006 (−0.038; 0.030)	−0.089 (−0.418; 0.234)
TC, mmol/l	−0.030 (−0.077; 0.016)	−0.151 (−0.478; 0.164)	−0.060 (−0.104; 0.087)	−0.104 (−1.083; 0.895)
HDL-C, mmol/l	−0.013 (−0.064; 0.038)	−0.180 (−0.546; 0.184)	0.001 (−0.123; 0.121)	−0.552 (−1.803; 0.664)
LDL-C, mmol/l	−0.049 (−0.119; 0.024)	−0.321 (−0.819; 0.187)	−0.006 (−0.171; 0.163)	−0.293 (−1.958; 1.384)
FFA, mmol/l	−0.017 (−0.038; 0.003)	−0.025 (−0.175; 0.130)	0.010 (−0.034; 0.061)	−0.052 (−0.473; 0.375)
TG, mmol/l	0.003 (−0.057; 0.063)	0.006 (−0.415; 0.452)	0.017 (−0.117; 0.152)	−0.023 (−1.313; 1.295)

Mean MVPA/ST describe the average time participants spent in MVPA/ST across pregnancy. Slope MVPA/ST describes the change in MVPA/ST across pregnancy. Associations with neonatal anthropometrics and cord blood parameters were estimated using Bayesian multilevel analyses. Results were stratified by offspring sex in case significant interactions were detected. Analyses adjusted for: maternal education (low/medium/high), pre-pregnancy BMI (kg/m^2^, centred around the mean), smoking (yes/no), gestational age at birth (weeks, centred around the mean), parity (nulliparous/multiparous), randomisation (Vitamin D intervention yes/no, healthy eating intervention yes/no). Cord blood analyses additionally adjusted for mode of delivery (spontaneous/assisted vaginal/caesarean section). Significant associations in bold.

*3BHB* 3-β-hydroxybutyrate, *BMI* body mass index, *CI* credible interval, *FFA* free fatty acids, *FM%* fat mass percentage, *HDL-C* high-density lipoprotein cholesterol, *LDL-C* low-density lipoprotein cholesterol, *MVPA* moderate-to-vigorous physical activity, *ST* sedentary time, *TC* total cholesterol.

Sensitivity analyses with SSF instead of FM% showed similar results; while associations in the combined study sample did not reach significance, results in the analyses stratified by sex showed associations between higher mean MVPA and an increase in MVPA and lower SSF in male, but not female, offspring (see Supplementary Table S1). Sensitivity analyses including only neonates with anthropometrics measured within 48 h after birth resulted in similar estimates, albeit not all significant, likely due to the smaller sample size.

No significant associations were found between MVPA and ST and birthweight (see Table 4).

Higher mean ST during pregnancy was associated with lower C-peptide cord blood levels in females (−0.145 µg/l; 95% CI: −0.279 µg/l, −0.005 µg/l, see Table 4), but not in males. Associations between MVPA, ST and other cord blood parameters were not significant.

## Discussion

In this secondary analysis of the DALI study, we investigated the association between repeatedly measured physical activity levels and sedentary time during pregnancy and neonatal anthropometrics and cord blood parameters.

In this population of pregnant women with obesity, the vast majority decreased their activity levels (99.5% of participants) and concurrently increased the time spent sedentary throughout pregnancy (98.1% of participants). Women, who on average had higher MVPA levels throughout pregnancy, gave birth to males with lower adiposity, compared to those who on average spent less time in MVPA. Moreover, women with a larger decrease in MVPA levels during pregnancy gave birth to males with higher adiposity. No significant associations with birthweight were found with either MVPA or ST. Apart from a negative association between average ST during pregnancy and cord blood C-peptide in female offspring, maternal MVPA and ST were not associated with cord blood parameters in our study.

These findings are relevant for the future development of body composition and risk of chronic diseases later in life, as it has been shown that neonatal fat mass is a predictor of childhood adiposity and obesity [5, 6].

### Physical activity and neonatal adiposity

While several studies showed associations between prenatal PA levels and offspring size at birth [9, 32], including macrosomia, LGA and SGA, fewer considered neonatal body composition as outcome. This distinction is of great relevance as in the current study we only found an association between MVPA and neonatal adiposity, but not with birthweight. This is in agreement with other studies who also found associations with neonatal fat, but not with birthweight [33]. This finding might be explained by the fact that fat mass, although only making up around 14% of the total weight, accounts for 46% of the variation in birthweight [34].

### Changes in MVPA and ST across gestation

We found that larger decreases in MVPA levels across pregnancy were associated with higher FM% in male offspring. Even though our own study as well as others clearly describe how activity levels change during gestation [35], many studies do not consider changes in PA levels in association with neonatal outcomes. An Irish cohort study that did analyse changing PA levels during pregnancy found that a decrease in PA levels between 15 and 20 weeks of gestation was associated with increased neonatal adiposity [36]. However, changes in activity levels were only determined by a single questionnaire item during the first half of pregnancy, even though a recent meta-analysis found that PA in late rather than early pregnancy was associated with neonatal anthropometry [37].

### Sex differences

This is the first study highlighting sex differences in the associations between repeatedly measured MVPA during gestation and neonatal adiposity. Sex differences in fetal development have been observed previously, with male fetuses generally appearing to be more impacted by their environment than females [38]. This is in concordance with our findings showing that the association between MVPA and neonatal FM% was driven mainly by male offspring.

### Cord blood parameters

We further found a significant inverse association between mean ST and cord blood C-peptide in females only. This is in contrast to our expectations, as we previously found that more ST during pregnancy was associated with higher maternal glucose and insulin parameters [39]. In addition, the maternal glucose metabolism during pregnancy has been shown to be strongly, positively associated with cord blood C-peptide [40] and is known to mediate the association between maternal and offspring adiposity [41]. While we did not find significant associations between maternal PA levels and FM% in females in our study sample, this might be explained by the weak correlation between C-peptide and FM% (*ρ* = 0.14, data not shown). It might also be an indication that female neonates are more insulin resistant, as suggested previously [42]. Finally, we cannot exclude the possibility that the association between MVPA and C-peptide was a chance finding since the credible interval is very close to zero.

Associations between maternal PA levels and cord blood parameters have not been studied extensively. One study found a significant association between maternal MVPA and cord blood leptin [43], another study found an association with neonatal cord blood HDL-C, but not with other cord blood lipids [44]. While FM% and cord blood leptin were significantly correlated in our study (*ρ* = 0.31, *p* < 0.001, data not shown), we did not find a significant association between either MVPA or ST and cord blood leptin or with any other cord blood parameters. Potentially, our relatively small sample size might have been too limited to detect weak associations.

### Underlying pathways

The maternal glucose metabolism is highly associated with neonatal adiposity [40, 45], supporting Pedersen’s hypothesis which states that maternal hyperglycaemia is transported through the placenta to the fetus, inducing a hyperinsulinemic response and resulting in increased deposition of body fat [46, 47]. Furthermore, PA during pregnancy has been shown to be associated with reduced glucose levels and improved insulin sensitivity [48] and has been shown to reduce the odds of developing GDM [49]. Indeed, women who were more active during pregnancy showed improved insulin and glucose levels, and gave birth to neonates with lower adiposity [10]. In the DALI study we previously reported that higher MVPA was associated with lower maternal insulin secretion [39], which in late pregnancy was associated with lower sum of skin folds in male neonates [15]. This provides support for the hypothesis of the maternal glucose-insulin axis being involved in the pathway between MVPA and neonatal FM%. However, we previously observed that in women carrying a male fetus glucose, insulin, HOMA-IR, and insulin secretion were mainly associated with ST and not MVPA [39]. The absence of associations between ST and neonatal adiposity in this study may indicate involvement of other pathways yet to be uncovered.

Finally, we previously observed that the intervention effect of the DALI lifestyle trial was mediated through a reduction in self-reported sedentary behaviour [13], but did not find significant associations between device-measured ST and neonatal FM% in the current study. This might be partly explained by different study populations, but mostly by different PA measurement instruments, as correlations between device-measured PA and questionnaire data are generally low [50].

### Strengths and weaknesses

A major strength of the study is the longitudinal design of repeated physical activity measurements, which allowed us to combine multiple measurements per participant in one model to investigate both average levels, as well as changes in MVPA and ST throughout pregnancy. This is especially relevant, as we showed that MVPA and ST change significantly across gestation. Another strength is the device-based measurement of PA levels as opposed to questionnaire data. We also considered sex differences, as it has been shown that male and female offspring have different strategies of dealing with exposures of the intra-uterine environment [17]. Furthermore, the study’s pan-European context allows generalisation of the results to pregnant women with overweight or obesity in Europe.

A potential weakness is the use of skinfold measurements for the estimation of neonatal body composition, as opposed to more valid methods like dual energy X-ray absorptiometry (DXA). A study in neonates and infants however showed high correlations and acceptable errors between skinfold and DXA fat mass measurements [51].

In addition, in our study, we only investigated physical activity and sedentary behaviours, but did not consider other lifestyle behaviours such as maternal nutrition, which is also a factor associated with offspring body composition [52, 53]. Furthermore, as MVPA and ST were only measured during waking hours, no conclusions can be drawn concerning the full 24-h cycle.

Finally, the relatively small sample size could have prevented us from discovering small, but potentially relevant findings. Future research should therefore include a larger number of participants and also include lean pregnant women, to ensure results can be generalised to the whole population.

## Conclusion

In this longitudinal study of pregnant women with obesity we found that higher mean prenatal physical activity levels at moderate-to-vigorous intensity are associated with lower neonatal adiposity, while a decrease in maternal physical activity across gestation was associated with increased neonatal adiposity in male offspring. More time spent sedentary throughout pregnancy was associated with higher cord blood C-peptide levels in female offspring. As neonatal adiposity is highly predictive of obesity in childhood, these findings are relevant in the prevention of rising numbers of childhood obesity and adiposity and underlines the importance of promoting physical activity during pregnancy. However, the question remains whether higher maternal physical activity levels also have a positive impact on offspring body composition on the long term.

## Supplementary information

Supplementary Table S1

## Data Availability

The datasets generated during and/or analysed during the current study are available from the corresponding author on reasonable request.
