# Supplementary material for: Physical activity and sedentary time across pregnancy and associations with neonatal weight, adiposity and cord blood parameters: a secondary analysis of the DALI study
Source: Int J Obes (Lond). 2023 Jul 27;47(9):873–81. doi: 10.1038/s41366-023-01347-9 (PMC10439006; doi:10.1038/s41366-023-01347-9)
Supplement: Supplementary file 1 — Supplementary Table S1 [file 41366_2023_1347_MOESM1_ESM.docx]

**Table S1: Sensitivity analyses of associations between maternal MVPA and ST and neonatal anthropometrics**

|  | **Mean MVPA (10 min)** | **Slope MVPA (min/week)** | **Mean ST (10% wear time)** | **Slope ST (% wear time/week)** |
| --- | --- | --- | --- | --- |
|  | *Estimate (95% CI)* | *Estimate (95% CI)* | *Estimate (95% CI)* | *Estimate (95% CI)* |
| *Sensitivity analysis 1:* |  |  |  |  |
| Neonatal fat mass % | **-**0.323 (-0.692; 0.051) | -2.111 (-4.575; 0.350) | -0.135 (-0.986; 0.719) | -5.323 (-13.059; 2.357) |
| Males | -0.487 (-1.000; 0.032) | **-4.165 (-7.480, -0.715)** | 0.805 (-0.390; 2.028) | -6.525 (-16.888; 3.658) |
| Females | -0.152 (-0.715; 0.379) | 0.012 (-3.506; 3.539) | -0.766 (-1.897; 0.531) | -2.124 (-13.628; 8.696) |
| *Sensitivity analysis 2:* |  |  |  |  |
| Neonatal SSF (mm) | -0.399 (-0.914; 0.112) | -2.272 (-5.592; 1.097) | 0.005 (-1.056; 1.111) | -4.821 (-15.000; 5.414) |
| Males | **-0.514 (-1.007; -0.019)** | **-4.594 (-7.834; -1.328)** | 0.704 (-0.492; 1.889) | -4.654 (-14.527; 5.208) |
| Females | -0.199 (-0.783; 0.380) | 0.138 (-3.348; 3.753) | -0.789 (-1.959; 0.414) | -3.852 (-14.653; 7.017) |

CI: credible interval; MVPA: moderate-to-vigorous physical activity; SSF: sum of skinfolds; ST: sedentary time.

Sensitivity analysis 1: Excluding those with measurements > 48 hours after birth (n = 180). Sensitivity analysis 2: Analyses repeated with SSF as outcome instead of FM% (n = 193).

Mean MVPA/ST describe the average time participants spent in MVPA/ST across pregnancy. Slope MVPA/ST describes the change in MVPA/ST across pregnancy. Associations with neonatal anthropometrics and cord blood parameters were estimated using Bayesian multilevel analyses. Analyses adjusted for: maternal education (low/medium/high), pre-pregnancy BMI (kg/m^2^, centred around the mean), smoking (yes/no), gestational age at birth (weeks, centred around the mean), parity (nulliparous/multiparous), randomisation (Vitamin D intervention yes/no, healthy eating intervention yes/no). Neonatal SSF analyses additionally adjusted for age at SSF measurement (hours). Significant associations in bold.
